# The Gut-Brain Axis as a Mediator of Environmental Endocrine Disruptors in Attention-Deficit/Hyperactivity Disorder: A Systematic Review and Mechanistic Synthesis

**DOI:** 10.1016/j.bpsgos.2026.100717

**Published:** 2026-03-06

**Authors:** Hui Wu, Meiqiao Yu, Shuo Huang, Yanghua Peng, Guo Wei, Congfu Huang

**Affiliations:** a Child Healthcare Department, Maternal and Child Health Hospital of PanYu District, Hexian Memorial Hospital of PanYu District, Guangzhou, China; b Department of Pediatrics, Longgang District Maternity & Child Healthcare Hospital of Shenzhen City (Affiliated Shenzhen Women and Children's Hospital [Longgang] of Shantou University Medical College), Medical Research Institute of Maternal and Child, Shenzhen, China

**Keywords:** Attention-deficit/hyperactivity disorder, Endocrine-disrupting chemicals, Gut-brain axis, Microbiome, Neurodevelopment, Systematic review

## Abstract

The rising global prevalence of attention-deficit/hyperactivity disorder (ADHD) underscores the importance of environmental factors, particularly environmental endocrine-disrupting chemicals (EEDs), whose mechanistic links to ADHD remain unclear. The gut-brain axis, a key modulator of neurodevelopment, is susceptible to EEDs and is altered in ADHD, suggesting a potential mediating pathway. Following Preferred Reporting Items for Systematic Reviews and Meta-Analyses (PRISMA) guidelines and PROSPERO registration (CRD420251152480), we systematically searched PubMed, Web of Science, and Embase (January 2014–July 2025) for studies on EEDs, gut microbiota, and ADHD. Data from 127 included studies (observational, experimental, interventional) were narratively synthesized to evaluate the gut-brain axis as a mediator. We found 1) consistent epidemiological associations between prenatal/childhood EED exposure (e.g., phthalates, bisphenol A, pesticides) and increased ADHD risk; 2) a distinct gut microbial signature in ADHD featuring reduced alpha diversity, elevated Firmicutes/Bacteroidetes ratio, depletion of beneficial taxa (*Lactobacillus*, *Bifidobacterium*), and impaired short-chain fatty acid (SCFA) production; 3) evidence that EED exposure induces convergent gut dysbiosis; and 4) interventional studies indicating that modulating the microbiota (via probiotics, synbiotics, fecal microbiota transplantation) can ameliorate ADHD-related behaviors. These findings support a novel mechanistic model wherein EEDs disrupt gut microbiota homeostasis, thereby contributing to ADHD pathogenesis via immune-inflammatory, microbial metabolite (e.g., SCFA), and neuroendocrine pathways along the gut-brain axis. This review synthesizes evidence positioning the gut-brain axis as a critical mediator linking EED exposure to ADHD. It proposes a unifying etiological framework and highlights the microbiome as a promising target for preventive and therapeutic strategies. Future longitudinal and intervention studies are needed to establish causality.

## Plain Language Summary

This systematic review proposes a novel mechanism linking environmental endocrine-disrupting chemicals (EEDs), such as phthalates and bisphenol A, to attention-deficit/hyperactivity disorder (ADHD). Evidence suggests that early-life EED exposure may disrupt the gut microbiome, leading to dysbiosis, which in turn could contribute to ADHD symptoms via immune, metabolic, and neuroendocrine pathways. The findings highlight the gut-brain axis as a key mediator and suggest microbiota-targeted interventions as promising future strategies for ADHD prevention and therapy.

Attention-deficit/hyperactivity disorder (ADHD) is a prevalent neurodevelopmental disorder characterized by inattention, hyperactivity, and impulsivity. Its global prevalence among children and adolescents has risen significantly, posing a major public health challenge (1,2). While genetic factors contribute substantially to ADHD etiology (3, 4, 5), they cannot fully explain the rising incidence, underscoring the critical role of environmental exposures, particularly during sensitive neurodevelopmental windows.

Environmental endocrine-disrupting chemicals (EEDs), such as bisphenol A (BPA), phthalates, pesticides, and persistent organic pollutants (e.g., polybrominated diphenyl ethers [PBDEs], polychlorinated biphenyls [PCBs]), are widespread exogenous compounds that interfere with hormonal signaling (6, 7, 8, 9). Exposure occurs primarily through dietary, consumer product, and environmental sources, with prenatal and childhood periods representing critical windows of vulnerability (10,11). Epidemiological studies have consistently linked early-life EED exposure to an increased risk of ADHD symptoms and diagnosis (12, 13, 14, 15, 16, 17).

The gut microbiota communicates bidirectionally with the central nervous system via the gut-brain axis, modulating neurodevelopment, behavior, and immune function (18,19). Individuals with ADHD exhibit distinct gut microbial dysbiosis, including reduced diversity, altered Firmicutes/Bacteroidetes (F/B) ratio, and decreased beneficial taxa such as *Lactobacillus* and *Bifidobacterium* (20, 21, 22, 23). Notably, EED exposure can also induce gut dysbiosis and barrier dysfunction (24, 25, 26, 27). This parallel effect raises the possibility of a shared pathway involving the gut-brain axis in the etiology of ADHD.

Although the associations between EEDs and ADHD, gut microbiota alterations in ADHD, and EED-induced dysbiosis have been investigated separately, no systematic review has integrated these 3 domains to elucidate the mediating role of the gut-brain axis. To address this gap, we conducted the first systematic review and mechanistic synthesis examining whether the gut-brain axis serves as a critical pathway linking EED exposure to ADHD pathogenesis. Our work proposes a novel theoretical framework and highlights the microbiota as a potential target for preventive and therapeutic strategies.

## Methods and Materials

This systematic review was conducted in accordance with the Preferred Reporting Items for Systematic Reviews and Meta-Analyses (PRISMA) guidelines and was prospectively registered on PROSPERO (Registration No. CRD420251152480). The primary objective was to systematically identify, evaluate, and synthesize the existing evidence regarding the potential mediating role of the gut-brain axis in the relationship between exposure to EEDs and ADHD.

### Search Strategy

We systematically searched PubMed, Web of Science Core Collection, and Embase for literature published from January 2014 to July 2025. The search strategy was constructed based on the PICO framework, combining medical subject heading terms and free-text keywords for the core concepts: ADHD, endocrine-disrupting chemicals, gut microbiota, and the gut-brain axis. No language or publication status restrictions were applied initially. Additionally, reference lists of included studies and relevant reviews were manually screened to identify further eligible publications. The complete search strings for each database are provided in Table S1.

### Eligibility Criteria

Eligible studies were original research reports investigating the relationships between EED exposure, gut microbiota, and ADHD. Included designs encompassed observational (cohort, case-control, cross-sectional), experimental (in vivo animal models), and interventional studies (randomized and nonrandomized trials). Human studies were restricted to children and adolescents (age ≤ 18 years), while animal studies required the use of established ADHD-relevant models. Eligible exposures involved the assessment or experimental administration of ≥1 EEDs. Required outcomes included 1) ADHD diagnosis or validated symptom scores, 2) gut microbiota composition/function data, or 3) measures of gut-brain axis intermediates (e.g., inflammatory markers, microbial metabolites, neuroendocrine parameters). We excluded non-original research, conference abstracts without full data, studies with unavailable or insufficient data, and studies focused solely on adults or non-ADHD animal models.

### Study Selection and Data Extraction

The study selection process was carried out independently by 2 reviewers to minimize bias. Initially, duplicate records identified across the different databases were removed using EndNote X9 software (Clarivate Analytics). Subsequently, the titles and abstracts of the remaining unique records were screened against the eligibility criteria. The full texts of articles deemed potentially relevant were then retrieved and assessed in detail for final inclusion. Any disagreements between the reviewers at any stage of the selection process were resolved through discussion or, if necessary, by consulting a third reviewer to reach a consensus.

A standardized, prepiloted data extraction form was used to collect relevant information from each included study. The extracted data included first author; publication year; country; study design; sample size and characteristics (e.g., age, sex); methods of EED exposure assessment (including the biomonitoring matrix and analytic technique); methodology for microbiota analysis (e.g., 16S ribosomal RNA [rRNA] gene sequencing, metagenomics, metabolomics); key statistical methods used; and primary findings related to the associations between EEDs, gut microbiota, gut-brain axis parameters, and ADHD outcomes. Specific data regarding effect sizes, confidence intervals, the direction and magnitude of microbial changes, and exposure levels were extracted where available.

### Quality Assessment (Risk of Bias)

The methodological quality and risk of bias of the included studies were critically appraised using standardized tools appropriate to each study design. First, observational studies (cohort, case-control) were evaluated using the Newcastle-Ottawa Scale (NOS). Second, cross-sectional studies were assessed using the checklist from the Agency for Healthcare Research and Quality. Third, animal studies were evaluated using the SYRCLE risk of bias tool. Finally, randomized controlled trials (RCTs) were assessed using the Cochrane RoB 2 tool.

Two reviewers independently conducted the quality assessments for each study. Any discrepancies in scoring were resolved through consensus discussion. The overall quality of evidence from the included studies was judged to be moderate to high for synthesis purposes (e.g., NOS score ≥6, SYRCLE score ≥5, majority of RCTs with low to moderate risk of bias).

### Data Synthesis and Integration

Given substantial heterogeneity across studies in exposures, outcomes, and methodologies, a quantitative meta-analysis was not feasible. Therefore, we conducted a narrative synthesis and a structured qualitative sensitivity analysis to evaluate the robustness of findings across methodological dimensions (see Supplemental Methods, section S1 for detailed rationale and approach) and organizing evidence into prespecified thematic categories: 1) EED exposure and ADHD risk, 2) gut microbiota in ADHD, 3) EED effects on the microbiota, and 4) mechanistic pathways involving the gut-brain axis.

To achieve a mechanistic synthesis, we adopted a structured, multistep approach to integrate evidence across study types and construct a theoretical model. This involved 1) assessing the evidence for the 3 core pairwise associations (EED-ADHD, ADHD-microbiota, EED-microbiota); 2) identifying overlapping microbial signatures between ADHD and EED-exposed states; 3) evaluating interventional studies supporting a causal or mediating role of the microbiota; and 4) synthesizing evidence on specific intermediate pathways (immune-inflammatory, microbial metabolite, neuroendocrine) to propose an integrated model of the gut-brain axis as a mediator. The synthesis included a critical appraisal of evidence quality and limitations. Further details on the synthesis rationale and a qualitative sensitivity analysis addressing methodological heterogeneity are provided in Supplemental Methods, section S1.

All cited studies were conducted in accordance with internationally recognized ethical standards, including the Declaration of Helsinki for human research and the ARRIVE (Animal Research: Reporting of In Vivo Experiments) guidelines for animal studies. Ethical approvals were obtained in the original studies as reported.

## Results

### Study Selection

The systematic database search identified 3582 records. After the removal of duplicates, 2543 unique articles underwent title and abstract screening. Following this, 558 full-text articles were assessed for eligibility, resulting in the inclusion of 127 studies for qualitative synthesis. The included studies comprised 78 observational studies, 36 experimental (animal) studies, and 13 interventional studies. The study selection process is detailed in the PRISMA flow diagram (Figure S1).

### Association Between EED Exposure and ADHD Risk

Consistent epidemiological evidence links early-life exposure to specific classes of EEDs with an elevated risk of ADHD or related traits. Prenatal exposure to phthalates (14,15) and BPA (28) has been associated with increased hyperactivity and externalizing behaviors in children. Childhood exposure to phthalates, phenols, and pesticides has been correlated with ADHD-related behaviors in cross-sectional analyses (10,13). Furthermore, prenatal exposure to persistent organic pollutants (e.g., PBDEs, PCBs, per- and polyfluoroalkyl substances [PFASs]) has been linked to an increased risk of ADHD diagnosis in prospective birth cohorts (12,29). While most studies have focused on single chemicals, emerging mixture analyses also support these associations (13,30). Detailed characteristics and findings of these epidemiological studies are summarized in Table 1.

Heterogeneity existed across studies, stemming from differences in exposure assessment methods, diagnostic tools, and control for confounders. While real-world exposure involves complex mixtures of chemicals, most studies assessed single chemicals, thereby limiting the understanding of combined effects (30). Nonetheless, most studies maintained significant associations after adjusting for key covariates.

Notably, emerging evidence suggests that potential differences in ADHD presentation related to the timing of EED exposure. Epidemiological evidence suggests that prenatal exposure to certain EEDs (e.g., phthalates, BPA) is associated with increased hyperactivity and externalizing behaviors in offspring (14,15), possibly reflecting disruption of early neurodevelopmental programming. The association between childhood exposure and specific ADHD symptom domains is less clear, although some cross-sectional studies have reported links between concurrent exposure and attentional problems (10). It has been hypothesized that exposure during later developmental periods, such as childhood and adolescence, may disproportionately affect neural circuits underlying attention and executive function, but direct longitudinal comparisons spanning prenatal and postnatal windows are needed to test this hypothesis (10,17). However, direct comparisons are constrained by variations in exposure assessment timing, diagnostic tools, and age at outcome assessment across studies. Future research utilizing life-course exposure models is needed to delineate critical and sensitive windows for specific EEDs and ADHD symptom domains.

### Gut Microbiota Alterations in Individuals With ADHD

Multiple studies have reported distinct gut microbial compositions in individuals with ADHD compared with control individuals (Table 2). The most commonly reported alterations, as detailed in the Table 2, include reduced alpha diversity, an elevated F/B ratio, and a decreased abundance of beneficial taxa such as *Lactobacillus* and *Bifidobacterium* (20,22,23). Metagenomic and metabolomic studies further point to more specific dysfunctions, including a reduction in *Bacteroides ovatus*, impairments in microbial genes related to short-chain fatty acid (SCFA) synthesis, and concomitant decreases in fecal SCFA levels alongside elevated inflammatory markers (22,31).

**Table 1:** Summary of Human Epidemiological Studies on the Association Between Prenatal and Childhood EED Exposure and ADHD Risk

Country	Study Design	Sample Size	EED Type(s)	Exposure Assessment	ADHD Assessment Tool	Key Findings/Notes	References
Denmark	Prospective cohort	1200	Phthalates	Maternal urine	CBCL	Prenatal DEHP exposure positively associated with hyperactivity	(15)
United States	Cross-sectional	450	BPA, parabens	Adolescent urine	Conners scale	BPA associated with inattention; parabens showed weaker association	(10)
Norway	Birth cohort	2000	PBDEs, PCBs, PFASs	Maternal/cord blood	ADHD diagnosis	Multiple POPs associated with increased ADHD risk	(12)
United States	Prospective cohort	500	Phthalates	Maternal urine	CPRS-R	Perinatal phthalate exposure linked to higher ADHD symptom scores	(14)
United States	Cross-sectional	700	Multiple EEDs	Adolescent urine	DSM-5	Mixture of phthalates, phenols, and pesticides correlated with ADHD behaviors	(13)
United States	Prospective cohort	1500	Pesticides, phenols	Maternal urine	CBCL	Prenatal pesticide and phenol exposure associated with increased ADHD risk	(40)

All listed studies adjusted for key covariates such as child sex and socioeconomic status.

ADHD, attention-deficit/hyperactivity disorder; BPA, bisphenol A; CBCL, Child Behavior Checklist; CPRS-R, Conners’ Parent Rating Scale-Revised; EED, environmental endocrine-disrupting chemical; PBDE, polybrominated diphenyl ether; PCB, polychlorinated biphenyl; PFAS, per- and polyfluoroalkyl substance; POP, persistent organic pollutant.

**Table 2:** Summary of Studies on Gut Microbiota Characteristics in Patients With ADHD

Country	Study Design	Sample Size, Case/Control, *n*	Microbiota Analysis	Key Findings	References
China	Cross-sectional	60/60	16S rRNA	↓ alpha diversity, ↑ F/B ratio	(23)
China	Cross-sectional	40/40	Metagenomics	↓ *Bacteroides ovatus*, ↓ SCFA synthesis genes	(22)
Denmark	Cross-sectional	80/80	16S rRNA	Distinct beta diversity between ADHD and ASD	(21)
Spain	Cross-sectional	30/30	16S rRNA	Significant beta diversity differences	(56)
Thailand	Cross-sectional	50/50	16S rRNA	↓ *Lactobacillus*, ↓ *Bifidobacterium*	(20)
China	Cross-sectional	70/70	Metagenomics + metabolomics	↓ SCFAs, ↑ inflammatory markers	(31)

ADHD, attention-deficit/hyperactivity disorder; ASD, autism spectrum disorder; F/B, Firmicutes/Bacteroidetes; rRNA, ribosomal RNA; SCFA, short-chain fatty acid.

### Effects of EED Exposure on the Gut Microbiota

Evidence from both human and animal studies indicates that EED exposure can significantly alter the gut microbiota (Table 3). In humans, exposure to EEDs such as phthalates and BPA is associated with reduced microbial diversity, a decrease in beneficial taxa (e.g., *Bifidobacterium*), an increase in potentially harmful taxa (e.g., *Enterobacteriaceae*), and markers of intestinal barrier dysfunction (10,25,32,33). Preclinical models consistently support these findings, showing that gestational or maternal exposure to diverse EEDs (e.g., pesticides, PBDEs) induces gut dysbiosis, characterized by similar reductions in diversity, shifts in community composition, and compromised gut barrier integrity in offspring (24,26,27). These convergent alterations across different EED classes and species suggest a robust potential for EEDs to disrupt gut microbial homeostasis.

**Table 3:** Summary of Experimental Studies on the Effects of EED Exposure on Gut Microbiota

Country	Study Type	Sample/Model	EED Type(s)	Exposure Route	Key Microbiota Changes	References
Spain	Human cross-sectional	Children	BPA, phthalates	Urinary biomonitoring	↓ *Bifidobacterium*, ↑ Enterobacteriaceae	(10,25)
United States	Human cross-sectional	Adolescents	BPA	Urinary biomonitoring	Altered composition, ↑ zonulin	(10)
Germany	Animal study	Mice	Glyphosate	Maternal drinking water	↓ diversity, ↑ inflammatory taxa	(26)
United States	Animal study	Rats	PBDEs	Gestational gavage	Dysbiosis, gut barrier disruption	(24)
China	Animal study	Rats	Antihypertensive drugs (EED mimicking)	Gestational exposure	Offspring dysbiosis, behavioral abnormalities	(27)

BPA, bisphenol A; EED, environmental endocrine-disrupting chemical; PBDE, polybrominated diphenyl ether.

**Table 4:** Summary of Intervention Studies Targeting the Gut-Brain Axis in ADHD or EED-Exposed Models

Study Type	Population/Model	Intervention	Key Microbiota Outcomes	Key Behavioral/Neurodevelopmental Outcomes	References
RCT (Human)	Children with ADHD	Synbiotic	↑ Microbial stability; ↑ SCFA-producing taxa	↓ ADHD symptoms (inattention, hyperactivity); improved daily functioning	(39)
RCT (Human)	Children and adults with ADHD	Synbiotic	Modulated plasma immune markers; ↑ SCFA levels	NA (primary outcomes: physiological)	(38)
RCT (Human)	Children with ADHD	*Bifidobacterium bifidum*	↑ Abundance of *Bifidobacterium*	↓ ADHD rating scale scores; improved social skills	(35)
RCT (Human)	Children/adolescents with ADHD	Probiotic adjunct to methylphenidate	Improved gut microbiota composition	Greater improvement in ADHD symptoms vs. placebo + methylphenidate	(36)
RCT (Human)	Children/adolescents with ADHD	*Lactobacillus acidophilus LB*	NA (gut microbiota not assessed)	Significant reduction in ADHD-RS total and subscores	(37)
Animal Trial	ADHD-like model rats	FMT	Transfer of donor microbiota	FMT from ADHD model donors recapitulated behavioral abnormalities	(34)
Animal Trial	PBDE-exposed rat offspring	Maternal probiotic supplementation	Attenuated PBDE-induced dysbiosis	Protected against social deficits and hyperactive behavior	(24)

ADHD, attention-deficit/hyperactivity disorder; ADHD-RS, ADHD Rating Scale; EED, environmental endocrine-disrupting chemical; FMT, fecal microbiota transplantation; NA, not available/not assessed; PBDE, polybrominated diphenyl ether; RCT, randomized controlled trial; SCFA, short-chain fatty acid.

## Discussion

### Synthesis of Evidence Supporting the Mediating Role of the Gut-Brain Axis

Our systematic review identified 3 robust pairwise associations: between 1) EED exposure and ADHD risk, 2) ADHD and gut dysbiosis, and 3) EED exposure and gut dysbiosis. To evaluate the overarching hypothesis that the gut-brain axis mediates the EED-ADHD link, we integrated this evidence, revealing 2 key convergent lines of support.

First, we observed convergent gut microbiota perturbations in both ADHD and EED-exposed states. These shared signatures include reduced alpha diversity, depletion of beneficial taxa such as *Bifidobacterium* (10,20,25), and, more specifically, a reduction in *Bacteroides ovatus* accompanied by impaired SCFA synthesis capacity (22,31). This overlap suggests a common microbial substrate that may be disrupted by EEDs and contribute to ADHD pathophysiology.

Second, intervention studies provide direct, although preliminary, support for the microbiota’s functional role. Fecal microbiota transplantation (FMT) from ADHD model rats recapitulates behavioral abnormalities in recipients (34), demonstrating phenotype transmissibility. Conversely, interventions that restore microbial homeostasis—including supplementation with specific probiotics [e.g., *Bifidobacterium bifidum* (35), *Lactobacillus acidophilus LB* (36,37)] or synbiotics (38,39)—ameliorate ADHD symptoms and related physiology. Notably, maternal probiotic supplementation can mitigate both PBDE-induced dysbiosis and associated neurobehavioral deficits in offspring (24). Collectively, these studies bolster the biological plausibility of the gut microbiota as a mediator between EED exposure and ADHD.

### Summary of Key Findings and Proposed Theoretical Framework

This systematic review synthesizes current evidence supporting the hypothesis that exposure to EEDs may contribute to ADHD pathogenesis, potentially via disruptions in the gut-brain axis. We identified consistent epidemiological associations linking prenatal and childhood exposure to various EEDs—including phthalates, BPA, pesticides, and persistent organic pollutants—with an elevated risk of ADHD or related behavioral symptoms (10,12,14,15,17,40). Concurrently, individuals with ADHD exhibit distinct gut microbial dysbiosis, characterized by reduced alpha diversity, an elevated F/B ratio, depletion of beneficial taxa such as *Lactobacillus* and *Bifidobacterium*, and impaired microbial metabolic functions, including reduced SCFA synthesis (20,22,23,31). Critically, evidence from both human and animal studies indicates that EED exposure can directly induce similar gut microbiota alterations (10,24, 25, 26), and intervention studies suggest that these microbial changes may influence neurodevelopment (24,34,35,38,39).

**Figure 1:**
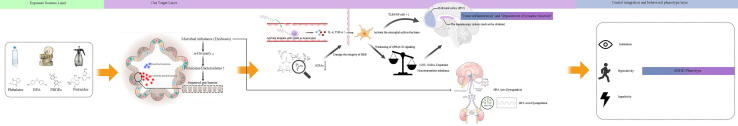
A proposed model depicting the gut-brain axis as a potential mediator between EED exposure and ADHD pathogenesis. This schematic illustrates a hypothesized mechanistic framework. Evidence suggests that prenatal and childhood exposure to EEDs (e.g., phthalates, BPA, pesticides, PBDEs) may disrupt gut microbiota homeostasis, leading to dysbiosis characterized by reduced microbial diversity, decreased beneficial taxa (e.g., *Lactobacillus*, *Bifidobacterium*), and increased proinflammatory species. This dysbiosis is proposed to contribute to ADHD-like behaviors through 3 interconnected pathways—1) immune-inflammatory pathway: EEDs may compromise intestinal integrity, potentially facilitating lipopolysaccharide translocation, triggering systemic inflammation and microglia-mediated neuroinflammation, which could impair prefrontal and dopaminergic development; 2) microbial metabolite pathway: EED-induced dysbiosis is associated with reduced production of SCFAs and may dysregulate neurotransmitter (e.g., GABA, serotonin, dopamine) balance, potentially disrupting BBB function and neuronal signaling; and 3) neuroendocrine pathway: EEDs and associated dysbiosis may jointly dysregulate the HPA axis, leading to aberrant cortisol rhythms, which may impair the development of brain regions critical for executive function and emotion regulation. Crosstalk between these pathways is hypothesized to potentially amplify the neurodevelopmental toxicity of EEDs. 5-HT, serotonin; ACTH, adrenocorticotropic hormone; ADHD, attention-deficit/hyperactivity disorder; BPA, bisphenol A; BBB, blood-brain barrier; CRH, corticotropin-releasing hormone; EED, environmental endocrine-disrupting chemical; GABA, gamma-aminobutyric acid; HPA, hypothalamic-pituitary-adrenal; IL-6, interleukin 6; LPS, lipopolysaccharide; NF-κB, nuclear factor-κB; PBDE, polybrominated diphenyl ether; SCFA, short-chain fatty acid; TNF-α, tumor necrosis factor α.

Based on this integrated evidence, we propose a conceptual model (Figure 1) depicting the gut-brain axis as a potential mediator in EED-associated ADHD. This framework hypothesizes that EEDs may influence neurodevelopment and behavior through several interconnected pathways:

1. Immune-inflammatory pathway: EEDs may compromise intestinal barrier integrity, potentially facilitating the translocation of bacterial products such as lipopolysaccharide (LPS) and triggering systemic and neuroinflammation, which could adversely affect prefrontal and dopaminergic systems (41, 42, 43, 44).
2. Microbial metabolite pathway: EED-induced dysbiosis is associated with reduced production of beneficial microbial metabolites, particularly SCFAs (22,31). Given the established role of SCFAs and other microbial metabolites in modulating the production and signaling of key neuroactive compounds (e.g., serotonin, GABA [gamma-aminobutyric acid], dopamine) within the gut-brain axis (41,45), EED-associated dysbiosis may thus disrupt neurochemical balance, contributing to ADHD pathophysiology. However, direct evidence linking EED-specific dysbiosis to altered neuroactive compound profiles in ADHD contexts remains an important area for future investigation.
3. Neuroendocrine pathway: EEDs and associated dysbiosis may jointly dysregulate the hypothalamic-pituitary-adrenal (HPA) axis, leading to aberrant cortisol rhythms that could impair the development of brain regions critical for executive function and emotion regulation (46, 47, 48).

Significant crosstalk between these pathways is plausible; for example, inflammation may suppress SCFA production and exacerbate HPA axis dysfunction (41,49), while HPA activation could further amplify gut permeability and systemic inflammation (47,48). Such potential positive feedback loops may amplify the neurodevelopmental impact of EEDs and contribute to the multidimensional symptoms of ADHD. Preclinical evidence further supports that specific probiotic strains can ameliorate hippocampal damage and memory deficits in ADHD models (50).

### Clinical and Public Health Implications

Our findings highlight the potential importance of reducing early-life EED exposure through public health policies aimed at limiting environmental levels of plasticizers, pesticides, and industrial chemicals found in consumer products (10, 11, 12). From a diagnostic perspective, microbial biomarkers (e.g., F/B ratio, SCFA levels) and inflammatory markers could be explored as potential future tools for early identification and stratification of ADHD subtypes (22,43,49). Therapeutically, interventions targeting the gut-brain axis—such as specific probiotics (e.g., psychobiotics), prebiotics, synbiotics, or dietary modifications—represent promising adjunctive strategies that may help alleviate ADHD symptoms by restoring microbial homeostasis and modulating host physiology (29,35,38,51,52). Several RCTs have reported that microbiota-directed interventions can improve behavioral and physiological outcomes in individuals with ADHD (35,38,39), supporting their potential integration into future treatment frameworks.

### Specificity of Microbial Alterations and the Imperative for Mixture-Based Approaches

#### Specificity of Gut Microbiota Signatures in ADHD

Gut microbiota dysbiosis is a recognized transdiagnostic feature across psychiatric and neurodevelopmental disorders (33,53). A critical question is whether the alterations observed in ADHD possess relative specificity. Emerging evidence suggests potential distinguishing patterns. For example, compared with autism spectrum disorder (ASD), ADHD appears more consistently associated with reductions in *Bacteroides ovatus* and impairments in microbial SCFA synthesis pathways (22,31). In contrast, ASD-related dysbiosis often involves more pronounced alterations in *Clostridia* species (21). However, these distinctions are primarily inferred from indirect comparisons across separate studies. Shared environmental risk factors, such as exposure to EEDs, may contribute to overlapping dysbiotic patterns, complicating the disentanglement of disorder-specific signatures. Therefore, direct, methodologically harmonized comparative studies profiling the gut microbiome across ADHD, ASD, and related conditions are urgently needed to definitively establish microbial specificity and elucidate shared versus unique pathophysiological mechanisms (a comparative overview of gut microbiota alterations across neuropsychiatric disorders is provided in Supplemental Discussion and Table 4).

#### Addressing the Complexity of Real-World EED Mixtures

Human exposure to EEDs invariably occurs as complex chemical mixtures, but most existing mechanistic evidence derives from single-chemical analyses (13,30), limiting translational relevance. To bridge this gap, future research must prioritize analytic frameworks that model combined exposures. Promising epidemiological studies have begun using advanced mixture analysis methods. For example, weighted quantile sum (WQS) regression identified a mixture of phthalates, phenols, and pesticides—with phthalates as the largest contributors—significantly associated with ADHD-related behaviors (13). Similarly, Bayesian kernel machine regression (BKMR) is being used to assess the joint effects of multiple pollutants on neurodevelopment (54). These approaches are crucial for approximating real-world exposure scenarios, where additive or synergistic effects are plausible. We strongly encourage the adoption of such mixture-analysis frameworks (e.g., WQS, BKMR, exposure-wide association studies) in future longitudinal cohorts (see Supplemental Discussion, section S4.1 for a primer on these analytic methods). This will enable a more accurate assessment of how combined EED exposures influence the gut-brain axis and contribute to ADHD pathogenesis, moving the field beyond the limitations of single-chemical paradigms.

### Limitations and Future Directions

Our synthesis, while robust, has several limitations. The predominance of cross-sectional human studies (10,12,15) precludes drawing definitive causal inferences. Residual confounding by factors such as diet, medication, or lifestyle cannot be fully excluded. Methodological heterogeneity in microbiome analysis (e.g., 16S rRNA gene sequencing vs. metagenomics) complicates direct cross-study comparisons (22,23). Animal studies often utilize supraphysiological EED doses or simplified exposure scenarios, which may limit their direct translational relevance (see Supplemental Discussion, section S5 for an extended discussion on dose-translation challenges, model variability, and interspecies microbiome differences).

To advance this field and establish causality, future research should 1) use longitudinal birth cohorts with repeated biosampling and causal mediation analysis to delineate temporal dynamics (12,33); 2) utilize interventional designs such as FMT in gnotobiotic models to validate the causal role of specific microbial taxa or metabolites (e.g., SCFAs) (34,55); 3) conduct rigorously designed RCTs testing targeted microbiome interventions, coupled with multi-omics profiling, to clarify therapeutic mechanisms (35,37,50); and 4) apply advanced mixture-analysis frameworks to real-world exposure data to elucidate combined effects (13,30,54).

### Methodological Heterogeneity: Impact and Strategies for Harmonization

The synthesized evidence originates from studies with substantial methodological diversity. A critical appraisal of this heterogeneity is essential for interpreting findings and guiding future research.

#### Impact on Evidence Interpretation

Variations in key methodologies influence the consistency and biological specificity of the reported associations:

1. Exposure assessment: Studies utilizing repeated biomonitoring (e.g., serial prenatal/postnatal samples) have generally reported more robust EED-ADHD associations than those using single time-point measures, suggesting that exposure misclassification may attenuate effect estimates in cross-sectional designs (10,12,14,32). The predominant focus on single EEDs, despite real-world exposure to complex mixtures, obscures potential interactive effects (13,30).
2. Microbiome profiling: Observations of reduced alpha diversity and an elevated F/B ratio in ADHD are consistent across 16S rRNA gene sequencing and shotgun metagenomic studies (20,23). However, finer-resolution signatures (e.g., *Bacteroides ovatus* depletion) and functional impairments (e.g., in SCFA synthesis genes) are discernible only with metagenomics or metabolomics (22,31), limiting cross-study mechanistic integration.
3. ADHD phenotyping: Studies utilizing clinician-confirmed DSM/ICD diagnoses tend to identify more distinct microbial alterations compared with those relying solely on broad-spectrum behavioral rating scales, underscoring the importance of precise phenotyping for biomarker discovery (21,23,56).

#### Qualitative Synthesis and Sensitivity Considerations

Given the substantial heterogeneity precluding formal meta-analysis, we conducted a structured qualitative assessment with evidence stratified by the methodological dimensions mentioned above. This sensitivity appraisal reinforced the robustness of the core EED-ADHD association and shared dysbiosis features. Crucially, it highlighted that studies using repeated exposure measures, deep metagenomic profiling, and clinical diagnoses yielded more precise and mechanistically insightful results. A structured qualitative sensitivity analysis is detailed in Supplemental Methods, section S1.2.

#### Strategies for Future Harmonization

To enhance causal inference and reproducibility, future research should adopt more harmonized methodological approaches, including

1. Standardized exposure protocols: implement repeated biospecimen collection across neurodevelopmental windows and prioritize advanced mixture analysis (e.g., WQS regression, BKMR) in longitudinal cohorts (13,30,54).
2. Multi-omic microbiome characterization: integrate metagenomics, metabolomics, and metatranscriptomics with 16S sequencing to capture taxonomic, functional, and metabolic insights simultaneously (22,31,57).
3. Consensus phenotyping: use validated diagnostic interviews alongside dimensional assessments to create well-characterized cohorts.
4. Collaborative consortia: establish international networks with shared protocols to enable pooled analyses and accelerate discovery.

## Conclusions

This systematic review consolidates evidence supporting a novel etiological pathway for ADHD, wherein exposure to EEDs may contribute to disease risk by disrupting the gut-brain axis. We propose a unifying mechanistic model in which EED-induced gut dysbiosis perturbs neurodevelopment via intertwined immune-inflammatory, microbial metabolite (e.g., SCFA), and neuroendocrine pathways. These findings underscore the dual translational imperative of mitigating early-life EED exposure and exploring microbiota-targeted interventions as potential preventive or adjunctive therapeutic strategies for ADHD. Future research must use longitudinal and interventional designs to establish causality and refine this emerging paradigm linking environmental toxicants, the gut microbiome, and brain health.

## Supplementary Material

Supplemental Methods, Results, Discussion, Figure S1, and Table S1
