# Supplementary material for: The Gut-Brain Axis as a Mediator of Environmental Endocrine Disruptors in Attention-Deficit/Hyperactivity Disorder: A Systematic Review and Mechanistic Synthesis
Source: Biol Psychiatry Glob Open Sci. 2026 Mar 6;6(3):100717. doi: 10.1016/j.bpsgos.2026.100717 (PMC13101782; doi:10.1016/j.bpsgos.2026.100717)
Supplement: Supplemental Methods, Results, Discussion, Figure S1, and Table S1 [file mmc1.pdf]

SUPPLEMENTARY INFORMATION

The Gut-Brain Axis as a Mediator of Environmental Endocrine Disruptors in Attention-Deficit/ Hyperactivity Disorder: A Systematic Review and Mechanistic Synthesis

Wu *et al.*

1. Supplementary Methods and Results

S1. Detailed Search Strategy

The complete, line-by-line search strategies executed in each database are provided below to ensure reproducibility. Strategies were constructed using a combination of Medical Subject Headings (MeSH) and relevant free-text terms for the core concepts: ADHD, endocrine-disrupting chemicals, gut microbiota, and the gut-brain axis.

S1.1 PubMed/MEDLINE Search Strategy (Executed July 15, 2025)

Line	Query
#1	("Attention Deficit Disorder with Hyperactivity"[Mesh] OR "Attention Deficit Hyperactivity Disorder"[tiab] OR ADHD[tiab])
#2	("Endocrine Disruptors"[Mesh] OR "endocrine disrupting chemicals"[tiab] OR "Bisphenol A"[Mesh] OR BPA[tiab] OR phthalates[tiab] OR "Diethylhexyl Phthalate"[tiab] OR pesticides[tiab] OR "Polychlorinated Biphenyls"[Mesh] OR PCBs[tiab] OR "Polybrominated Diphenyl Ethers"[Mesh] OR PBDEs[tiab] OR "Perfluoroalkyl Substances"[Mesh] OR PFAS[tiab])
#3	("Gastrointestinal Microbiome"[Mesh] OR "gut microbiota"[tiab] OR "intestinal flora"[tiab] OR dysbiosis[tiab] OR "gut-brain axis"[tiab] OR "microbiome-gut-brain"[tiab] OR "short-chain fatty acids"[tiab] OR SCFA[tiab])
#4	#1 AND #2 AND #3
Filters:	Publication date: January 1, 2014 – July 15, 2025.

S1.2 Web of Science Core Collection Search Strategy (Executed July 15, 2025)

Line	Query
#1	TS=("attention deficit hyperactivity disorder" OR ADHD OR "hyperkinetic disorder")
#2	TS=("endocrine disruptor" OR "endocrine disrupting chemical" OR bisphenol A OR BPA OR phthalate OR pesticide OR "polychlorinated biphenyl" OR PCB OR "polybrominated diphenyl ether" OR PBDE OR "perfluoroalkyl substance" OR PFAS)
#3	TS=("gut microbiome" OR "intestinal microbiota" OR dysbiosis OR "gut-brain axis" OR "microbiome-gut-brain" OR "short chain fatty acid" OR SCFA)
#4	#1 AND #2 AND #3
Timespan:	January 1, 2014 – July 15, 2025.<br>Indexes: SCI-EXPANDED, SSCI, ESCI.<br>Language: All.

S1.3 Embase Search Strategy (via Ovid) (Executed July 16, 2025)

Line	Query
#1	'attention deficit hyperactivity disorder'/exp OR 'adhd':ti,ab,kw OR 'attention deficit

	disorder with hyperactivity':ti,ab,kw
#2	'endocrine disruptor'/exp OR 'endocrine disrupting chemical':ti,ab,kw OR 'bisphenol a'/exp OR 'bpa':ti,ab,kw OR 'phthalic acid derivative'/exp OR 'phthalate':ti,ab,kw OR 'pesticide'/exp OR 'pesticide':ti,ab,kw OR 'polychlorinated biphenyl'/exp OR 'pcb':ti,ab,kw OR 'polybrominated diphenyl ether'/exp OR 'pbde':ti,ab,kw OR 'perfluoroalkyl substance'/exp OR 'pfas':ti,ab,kw
#3	'gut microbiome'/exp OR 'intestinal microbiota':ti,ab,kw OR 'dysbiosis':ti,ab,kw OR 'gut brain axis'/exp OR 'gut-brain axis':ti,ab,kw OR 'short chain fatty acid'/exp OR 'short chain fatty acid':ti,ab,kw
#4	#1 AND #2 AND #3
Filters:	AND [2014-2025]/py<br>Language: No restriction.

S2. Supplementary Figure: PRISMA 2020 Flow Diagram

Figure S1. PRISMA 2020 flow diagram detailing the identification, screening, and inclusion of studies for the systematic review.

PRISMA 2020 flow diagram for new systematic reviews which included searches of databases and registers only

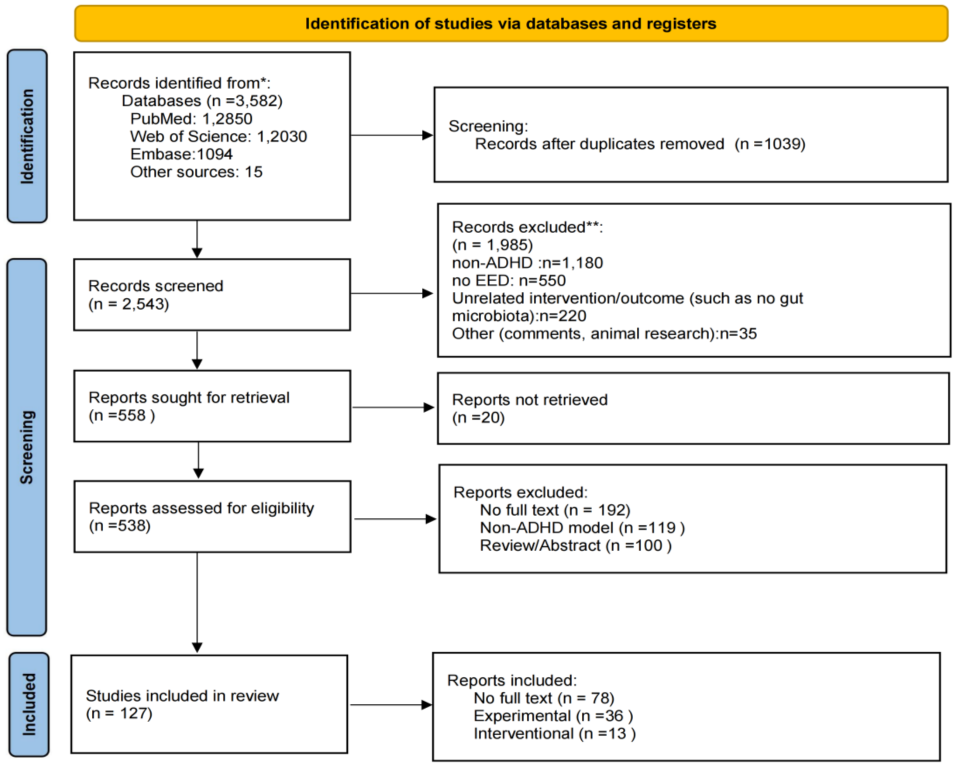

*Consider, if feasible to do so, reporting the number of records identified from each database or register searched (rather than the total number across all databases/registers).

**If automation tools were used, indicate how many records were excluded by a human and how many were excluded by automation tools.

Source: Page MJ, et al. BMJ 2021;372:n71. doi: 10.1136/bmj.n71.

This work is licensed under CC BY 4.0. To view a copy of this license, visit https://creativecommons.org/licenses/by/4.0/

Figure 1. PRISMA Flow Diagram of the Literature Search and Study Selection Process

This flowchart illustrates the systematic process of identifying, screening, and including studies for the current systematic review, which investigates the mediating role of the gut-brain axis in the relationship between environmental endocrine disruptors (EEDs) and ADHD. The search was conducted across three electronic databases (PubMed, Web of Science, and Embase) from inception to July 2025. A total of 3,582 records were identified, from which 127 studies met the predefined eligibility criteria and were included in the qualitative synthesis.

S3. Detailed Methodology for Narrative Synthesis and Qualitative Sensitivity Analysis

S3.1 Rationale for Adopting a Narrative Synthesis Approach

A formal quantitative meta-analysis was precluded due to substantial and irreducible

heterogeneity across the included studies (n=127). This heterogeneity manifested in three key domains: 1) Exposure Assessment: Varied biomonitoring matrices (urine, blood, cord blood), analytical techniques, and timing (single vs. repeated measures). 2) Microbiome Profiling: Use of different techniques (16S rRNA gene sequencing vs. shotgun metagenomics vs. metabolomics), targeting different genomic regions, and employing diverse bioinformatic pipelines. 3) ADHD Phenotyping: Utilization of different diagnostic criteria (DSM-IV, DSM-5), tools (clinical interview vs. parent/teacher rating scales), and age groups. Consequently, we employed a narrative synthesis approach, structured around pre-specified thematic domains (EED-ADHD, ADHD-microbiota, EED-microbiota, interventions), to integrate evidence and construct a mechanistic model, as recommended for complex interdisciplinary reviews.

S3.2 Framework for the Qualitative Sensitivity Analysis

To assess the robustness of our synthesized findings amidst methodological diversity, we conducted a structured qualitative sensitivity analysis. The process involved four steps:

1)Dimension Definition: We identified three primary sources of methodological heterogeneity: (i) Exposure assessment strategy, (ii) Microbiome profiling depth, and (iii) ADHD phenotyping precision.

2)Evidence Stratification: Each included study was categorized within these dimensions:

①Exposure: "Single/Cross-sectional" vs. "Repeated/Longitudinal" assessment.

②Microbiome: "16S rRNA gene sequencing" vs. "Shotgun Metagenomics/Metabolomics".

③Phenotyping: "Rating Scale only" vs. "Clinical Diagnosis confirmed".

3)Consistency Evaluation: For each core association (e.g., EED-ADHD), we examined whether the direction and significance of findings were consistent across studies within the same methodological stratum and across different strata.

4)Robustness Judgment: We evaluated whether the main synthesized conclusions held across methodological variations, noting where advanced methods yielded more precise or mechanistic insights.

S3.3 Key Observations from the Qualitative Sensitivity Analysis

This stratified appraisal reinforced the core conclusions while clarifying the impact of

methodology:

1)EED-ADHD Association: Studies using repeated exposure measures (e.g., prenatal & postnatal biospecimens) reported more robust and consistent effect estimates [1-3]. Associations in studies relying on single time-point measures were often attenuated but remained significant, suggesting exposure misclassification may bias results toward the null in cross-sectional designs.

2)Microbiota-ADHD Association: Observations of reduced alpha-diversity and an elevated F/B ratio were highly consistent across both 16S and metagenomic studies [4, 5], indicating these are robust, coarse-grained signatures. In contrast, finer-resolution signals like the depletion of Bacteroides ovatus and impairments in SCFA synthesis genes were exclusively and reliably detected in studies using shotgun metagenomics or metabolomics [6,7], underscoring the taxonomic resolution limit of 16S sequencing for detailed mechanistic insights.

3)Phenotyping Impact: Studies utilizing clinician-confirmed DSM/ICD diagnoses identified more specific and pronounced microbial alterations compared to those using broad-spectrum behavioral rating scales alone [5, 8, 9], highlighting the importance of precise phenotyping for biomarker discovery.

4)Overall Robustness: The tripartite relationship (EED→dysbiosis→ADHD risk) was supported across methodological approaches. However, the biological specificity and strength of mechanistic inferences were strongest in studies employing longitudinal exposure assessment, deep metagenomic profiling, and clinical diagnoses.

2.Supplementary Extended Discussion

S4. Extended Considerations on EED Mixture Effects and Disorder Specificity

S4.1 Analytical Approaches for Complex Mixture Exposures: A Primer

Human exposure to EEDs is to complex chemical mixtures, not isolated compounds. Advanced statistical frameworks are essential to model these real-world exposures:

1)Weighted Quantile Sum (WQS) Regression: This method identifies a weighted index of chemicals (the "WQS index") most associated with an outcome, useful when all chemicals are hypothesized to act in the same direction. It was effectively used by Shoaff et al. [10] to identify a phthalate-phenol-pesticide mixture linked to ADHD behaviors.

2)Bayesian Kernel Machine Regression (BKMR): BKMR flexibly models the joint,

potentially non-linear and interactive, effects of multiple exposures. It can identify critical components within a mixture and visualize exposure-response functions. Its application is growing in environmental epidemiology, as noted in studies like Stratmann et al. [11].

3)Exposure-Wide Association Studies (ExWAS): This agnostic, high-dimensional approach screens numerous exposures simultaneously, controlling for false discovery. It is valuable for hypothesis generation regarding which EEDs in a mixture warrant further investigation.

Future research must prioritize integrating these mixture-analysis frameworks into longitudinal birth cohorts to move beyond the limitations of single-chemical paradigms and better approximate the true complexity of developmental exposure.

S4.2 Comparative Gut Microbiota Profiles Across Neuropsychiatric Disorders: Current Evidence and Gaps

Gut dysbiosis is a transdiagnostic phenomenon. Table S1 summarizes reported microbial alterations across major neuropsychiatric conditions, highlighting potential areas of specificity and overlap.

Table S1. Comparative Overview of Gut Microbiota Alterations in Select Neuropsychiatric and Neurodevelopmental Disorders.

Disorder	Commonly Reported Alterations (Relative to Controls)	Potential Distinguishing Features vs. ADHD	Key References (Examples)
ADHD	↓ α-diversity, ↑ F/B ratio, ↓ Bifidobacterium, ↓ Lactobacillus, ↓ Bacteroides ovatus, ↓ SCFA production.	More consistent link to B. ovatus depletion and impaired SCFA synthesis pathways.	[4-7]
Autism Spectrum Disorder (ASD)	↓ α-diversity (often), ↑ Clostridium clusters, alterations in Bacteroidetes and Firmicutes, distinct metabolomic profiles (e.g., altered bile acids).	More pronounced alterations in specific Clostridia species; different metabolomic signatures.	[8]
Major Depressive Disorder (MDD)	↓ Faecalibacterium, ↓ microbial richness, altered tryptophan metabolism.	Prominent reduction in Faecalibacterium prausnitzii (a butyrate producer); stronger links to inflammation and tryptophan-serotonin pathway.	[12, 13]
Schizophrenia	↓ α-diversity, ↑ Lactobacillus (in some studies), altered microbial	Inconsistent directional changes for certain taxa;	[12]

	gene pathways for neurotransmitter synthesis.	potential unique viral or fungal components.	

Note: This table is synthesized from separate studies and should be interpreted with caution. Direct, methodologically harmonized case-control studies across disorders are urgently needed to confirm these comparative patterns and disentangle disorder-specific signatures from general markers of psychopathology or shared environmental risk factors (like EED exposure).

S5. Limitations of Animal Studies and Dose-Translation Considerations

While invaluable for mechanistic insight, the animal studies included in this review have limitations affecting translational interpretation:

1)Dose and Relevance: Many studies use supraphysiological doses or acute exposure regimens to elicit measurable effects within experimental timeframes [14-16]. This raises questions about the relevance to chronic, low-level human environmental exposure. Future preclinical work should prioritize environmentally relevant dose (ERD) studies that better mimic human exposure scenarios.

2)Model Variability: Different animal models of ADHD (e.g., spontaneously hypertensive rats, DAT knockout mice, pharmacologically-induced models) were used. Each model recapitulates different aspects of ADHD symptomatology (hyperactivity, inattention, impulsivity) and may involve distinct underlying neurobiology and gut microbiome responses, complicating direct comparison and generalization.

3)Microbiome Differences: The baseline gut microbiota of laboratory rodents differs substantially from humans in composition and function. While dysbiotic trends (e.g., reduced diversity) may be consistent, specific taxon-level changes observed in rodents may not directly translate to humans.

Acknowledging these limitations is crucial when integrating preclinical evidence into a human-focused mechanistic model. They underscore the need for translational research that bridges controlled animal studies with observational human data.

3.References

1.Lenters V, Iszatt N, Forns J, Čechová E, Kočan A, Legler J, et al. Early-life exposure to persistent organic pollutants (OCPs, PBDEs, PCBs, PFASs) and attention-deficit/hyperactivity disorder: A multi-pollutant analysis of a Norwegian birth cohort. *Environ Int* 2019;125:33-42. doi:

10.1016/j.envint.2019.01.020.

2.Watkins DJ, Meeker JD, Tamayo-Ortiz M, Sánchez BN, Schnaas L, Peterson KE, et al. Gestational and peripubertal phthalate exposure in relation to attention performance in childhood and adolescence. *Environ Res* 2021;196:110911. doi: 10.1016/j.envres.2021.110911.

3.Harigai W, Mikami K, Choudhury ME, Yamauchi H, Yajima C, Shimizu S, et al. Effects of fecal microbiota transplantation on behavioral abnormality in attention deficit hyperactivity disorder-like model rats. *J Pharmacol Sci* 2025;157(3):189-198. doi: 10.1016/j.jphs.2025.01.007.

4.Boonchooduang N, Louthrenoo O, Chattipakorn N, Chattipakorn SC. Possible links between gut-microbiota and attention-deficit/hyperactivity disorders in children and adolescents. *Eur J Nutr* 2020;59(8):3391-3403. doi: 10.1007/s00394-020-02383-1.

5.Wang N, Gao X, Zhang Z, Yang L. Composition of the Gut Microbiota in Attention Deficit Hyperactivity Disorder: A Systematic Review and Meta-Analysis. *Front Endocrinol (Lausanne)* 2022;13:838941. doi: 10.3389/fendo.2022.838941.

6.Li Y, Sun H, Huang Y, Yin A, Zhang L, Han J, et al. Gut metagenomic characteristics of ADHD reveal low *Bacteroides ovatus*-associated host cognitive impairment. *Gut Microbes* 2022;14(1):2125747. doi: 10.1080/19490976.2022.2125747.

7.Al-Saleh I, Aljerayed Y, Gheith M, Alobaid N, Alenazi H, Elkhatib R, et al. Longitudinal analysis of maternal exposure to phthalates and bisphenol A and their impact on infant neurodevelopment and autistic behavior: The potential mediating role of thyroid hormones. *Int J Hyg Environ Health* 2025;269:114647. doi: 10.1016/j.ijheh.2025.114647.

8.Bundgaard-Nielsen C, Lauritsen MB, Knudsen JK, Rold LS, Larsen MH, Hindersson P, et al. Children and adolescents with attention deficit hyperactivity disorder and autism spectrum disorder share distinct microbiota compositions. *Gut Microbes* 2023;15(1):2211923. doi: 10.1080/19490976.2023.2211923.

9.McGuinness AJ, Davis JA, Dawson SL, Loughman A, Collier F, O'Hely M, et al. A systematic review of gut microbiota composition in observational studies of major depressive disorder, bipolar disorder and schizophrenia. *Mol Psychiatry* 2022;27(4):1920-1935. doi: 10.1038/s41380-022-01456-3.

10.Shoaff JR, Coull B, Weuve J, Bellinger DC, Calafat AM, Schantz SL, et al. Association of Exposure to Endocrine-Disrupting Chemicals During Adolescence With

Attention-Deficit/Hyperactivity Disorder-Related Behaviors. *JAMA Netw Open* 2020;3(8):e2015041. doi: 10.1001/jamanetworkopen.2020.15041.

11.Kwak MJ, Kim SH, Kim HH, Tanpure R, Kim JI, Jeon BH, et al. Psychobiotics and fecal microbial transplantation for autism and attention-deficit/hyperactivity disorder: microbiome modulation and therapeutic mechanisms. *Front Cell Infect Microbiol* 2023;13:1238005. doi: 10.3389/fcimb.2023.1238005.

12.Wang LJ, Tsai CS, Chou WJ, Kuo HC, Huang YH, Lee SY, et al. Add-On *Bifidobacterium Bifidum* Supplement in Children with Attention-Deficit/Hyperactivity Disorder: A 12-Week Randomized Double-Blind Placebo-Controlled Clinical Trial. *Nutrients* 2024 ;16(14):2260. doi: 10.3390/nu16142260.

13.Stratmann M, Özel F, Marinopoulou M, Lindh C, Kiviranta H, Gennings C, et al. Prenatal exposure to endocrine disrupting chemicals and the association with behavioural difficulties in 7-year-old children in the SELMA study. *J Expo Sci Environ Epidemio*l 2024 Dec 19. doi: 10.1038/s41370-024-00739-x.

14.Denys ME, Kozlova EV, Liu R, Bishay AE, Do EA, Piamthai V, et al. Maternal probiotic supplementation protects against PBDE-induced developmental, behavior and metabolic reprogramming in a sexually dimorphic manner: Role of gut microbiome. *Arch Toxicol* 2025;99(1):423-446. doi: 10.1007/s00204-024-03882-4.

15.Buchenauer L, Haange SB, Bauer M, Rolle-Kampczyk UE, Wagner M, Stucke J, et al. Maternal exposure of mice to glyphosate induces depression- and anxiety-like behavior in the offspring via alterations of the gut-brain axis. *Sci Total Environ* 2023;905:167034. doi: 10.1016/j.scitotenv.2023.167034.

16.Li HB, Xu ML, Xia WJ, Dong YY, Peng B, Su Q, et al. Antihypertensive treatment during pregnancy induces long-term changes in gut microbiota and the behaviors of the attention deficit hyperactivity disorder offspring. *Toxicol Appl Pharmaco*. 2024;486:116946. doi: 10.1016/j.taap.2024.116946.
